# Validity of the Chinese Language Patient Health Questionnaire 2 and 9: A Systematic Review

**DOI:** 10.1089/heq.2022.0030

**Published:** 2022-08-18

**Authors:** Leena Yin, Semhar Teklu, Hallen Pham, Rocky Li, Peggy Tahir, Maria E. Garcia

**Affiliations:** ^1^ School of Medicine, University of California, San Francisco, San Francisco, California, USA.; ^2^ University of California, Berkeley, Berkeley, California, USA.; ^3^ Department of Internal Medicine, University of Washington, Seattle, Washington, USA.; ^4^ College of Osteopathic Medicine of the Pacific, Western University of Health Sciences, Pomona, California, USA.; ^5^ UCSF Library, University of California, San Francisco, San Francisco, California, USA.; ^6^ Division of General Internal Medicine, Department of Medicine, Center for Aging in Diverse Communities, University of California, San Francisco, San Francisco, California, USA.; ^7^ Multiethnic Health Equity Research Center, Division of General Internal Medicine, Department of Medicine, University of California, San Francisco, San Francisco, California, USA.; ^8^ Department of Epidemiology and Biostatistics, University of California, San Francisco, San Francisco, California, USA.

**Keywords:** depressive symptoms, Chinese, depression screening, language barriers, psychometrics

## Abstract

**Introduction:**

Chinese Americans with limited English proficiency have higher mental health needs than English speakers but are more likely to be undiagnosed and undertreated for depression. Increasing anti-Asian hate crimes during the COVID-19 pandemic has increased the urgency to accurately detect depressive symptoms in this community. This systematic review examines the validity of the Patient Health Questionnaire (PHQ)-2/9 for depression screening in Chinese-speaking populations.

**Methods:**

We queried PubMed, Web of Science, Embase, and PsycINFO databases, examining studies through September 2021. Studies were included if they evaluated the Chinese language PHQ-2 or PHQ-9 and diagnosed depression using a clinical interview. Two investigators independently extracted study data and assessed quality using the QUADAS-2. Study sensitivities and specificities were combined in random effects meta-analyses.

**Results:**

Of 513 articles, 20 met inclusion criteria. All examined the PHQ-9; seven also examined the PHQ-2. Studies were conducted in Mainland China (17), Hong Kong (1), Taiwan (1), and the United States (1). Fourteen studies were published in English; six in Chinese. Studies were diverse in setting, participant age, and comorbidities. For the Chinese language PHQ-9, Cronbach's alpha ranged from 0.765 to 0.938 for included studies (optimal cutoff scores ranged from 6 to 11). For the PHQ-2, Cronbach's alpha ranged from 0.727 to 0.785 (optimal cutoff scores 1–3). Overall, the PHQ-9 pooled sensitivity was 0.88 (95% CI 0.86–0.90), and pooled specificity was 0.87 (95% CI 0.83–0.91). Similarly, the pooled PHQ-2 sensitivity was 0.84 (95% CI 0.80–0.87), and pooled specificity was 0.87 (95% CI 0.78–0.93). The overall risk of bias was low (12 studies) or indeterminate (8 studies).

**Discussion:**

While limited by missing study information, the Chinese language PHQ-9 appears to be a valid depression screening tool among Chinese-speaking populations across geographic and clinical settings. Further research should explore optimal cutoff scores for this population for routine depression screening and the validity of the tool to measure response to depression treatment.

## Introduction

Depression is a major public health concern, which affects 19.4 million adults in the United States^1^ and 280 million people worldwide.^2^ Depression leads to poor quality of life,^3^ worse health outcomes with increased morbidity and mortality,^4,5^ and increased health care costs.^6^ While patients with limited English proficiency (LEP) are more likely to present with more severe depressive symptoms compared with English-only speakers,^7–9^ clinicians are less likely to diagnose these patients with depression.^10^ This exacerbates existing disparities in access to mental health care among individuals with LEP.^11–13^

Almost 3 million U.S. residents speak Chinese at home, making it the third most spoken language in the nation.^14^ Past studies have found that Chinese Americans with LEP have high mental health burden, with the prevalence of depressive symptoms among Chinese monolingual primary care patients in the United States as high as 20%; however, Asian patients in the United States face disparities in mental health care access and have lower odds of receiving needed services than patients from other ethnic groups.^13,15^

Furthermore, Asian patients with LEP who are able to access the health care system may find that their symptoms go unrecognized compared with their English-proficient counterparts.^16–19^ In fact, one study of English, Spanish, and Chinese-speaking primary care patients found that physicians were least likely to diagnose depressive symptoms in Chinese-speaking patients.^10^ Thus, one pathway to improving access to depression treatment and specialty mental health services for Chinese patients with LEP is ensuring that physicians are using evidence-based tools to better identify patients with depressive symptoms.

Of particular note, the heightened anti-Asian racism throughout the COVID-19 pandemic has been associated with an increase in depression and anxiety in the Asian American community, further highlighting the need for physicians to screen effectively for mental health symptoms in this population.^20^

The Patient Health Questionnaire (PHQ)-9 has long been recognized as an effective screening instrument for depression among English-proficient adults.^21^ It is commonly used in primary care settings as a first-line measure for detecting depressive symptoms in adults,^22^ as recommended by the U.S. Preventive Services Task Force.^23^ The PHQ-2 is a briefer version of the PHQ-9 with similar sensitivity but higher specificity when paired with the PHQ-9 to follow up on positive screens,^24^ which is commonly used due to its efficiency. While the original PHQ-9 was developed and validated in English, it has since been translated and used in many other languages, including Chinese.^25^ However, given that the presentation of depression can vary across cultures and languages,^26,27^(p.1) we must determine the validity of the PHQ-2 and PHQ-9 in Chinese languages.

Two systematic reviews of Chinese language depression screening tools have been previously conducted but both had limitations that affect generalizability to our population of interest. These reviews^28,29^ focused on a variety of screening tools, with fewer studies specifically evaluating the PHQ-9 (a maximum of four studies in one review). Additionally, both research teams excluded studies conducted outside of China, limiting their applicability to Chinese-speaking immigrants in the United States. Both reviews included studies that compared the PHQ-9 with a variety of different instruments, including more widely used research tools such as Center for Epidemiologic Studies Depression Scale; neither conducted a clinical interview as the gold standard for diagnosis of depression. Furthermore, the systematic review by Sun et al, which concluded that the PHQ-9 was “acceptable,” was published in Chinese only, and thus remains inaccessible to English-speaking clinicians who may wish to apply this evidence to their practice.

Chiu and Chin concluded that the PHQ-9 was sensitive and “highly effective” for screening for depression in Chinese primary care; however, they only looked at articles published in English, with only four studies included in final review. Since these reviews were conducted in 2016, multiple studies evaluating the PHQ-2 and PHQ-9 have been published, warranting re-evaluation of the evidence.

We conducted a systematic review of the current literature evaluating the validity of both the Chinese language PHQ-2 and PHQ-9 for depression screening, specifically in comparison to a clinical interview as the gold standard for diagnosing depression, across geographic and practice settings.

## Methods

### Publication search

To find relevant articles, we performed comprehensive searches in PubMed, Web of Science, Embase, and PsycINFO databases with a university librarian (author P.T.). Searches were developed around these concepts: the PHQ-2 and PHQ-9, screening for depression and depressive disorders, and the validity and efficacy of the questionnaires, with a focus on the tool in Chinese languages. We chose to include multiple spoken Chinese languages (Mandarin, Cantonese, etc.) as written traditional/simplified Chinese does not distinguish between them and the PHQs are usually administered in written form. We used multiple synonyms for the different concepts to create sensitive searches that would not miss any eligible articles. We used both index terms (Mesh, Emtree) and keywords to develop the searches, and limited the search in PsycINFO to peer-reviewed articles (because this database includes non-peer-reviewed sources such as news articles and dissertations).

The full search strategies for each database can be found in the search appendix (Appendix A1). The initial searches were performed in December 2020, and a search update was performed in September 2021 to capture any relevant studies in the interval period. We also searched reference lists of retrieved articles and systematic reviews for relevant articles.

### Study selection

Studies that met all of the following criteria were included in this systematic review: (1) participants were 16 years of age or older; (2) participants were primarily Chinese speakers of any language or dialect; (3) studies specifically examined either the PHQ-2 or PHQ-9; (4) questionnaire validity was studied for the purpose of screening specifically for major depression; (5) the questionnaire(s) studied were validated against a clinical interview as the gold standard for diagnosing depression; and (6) outcomes included biometric properties of the questionnaire(s).

We chose criterion five following best practices of diagnostic research, wherein validity studies should utilize the gold standard for comparison if one exists; in the field of psychiatry, the gold standard for depression diagnosis is the clinical interview, structured or semistructured, performed by a trained health professional or researcher. Examples include the Structured Clinical Interview for DSM (SCID), Mini-International Neuropsychiatric Interview (MINI), Composite International Diagnostic Interview (CIDI), or Schedules for Clinical Assessment in Neuropsychiatry (SCAN).^30–32^

We excluded studies on the basis of one or more of the following: (1) inappropriate population (e.g., children, English- or other non-Chinese language spoken); (2) studies conducting factor analysis alone; (3) inappropriate gold standard (i.e., studies evaluating the PHQ-2 or PHQ-9 against another clinical scale or questionnaire only); (4) studies examining other versions of the PHQ (e.g., PHQ-15 or PHQ-8); and (5) studies examining diagnosis or screening for disorders other than major depression, such as postpartum depression.

Two investigators (L.Y. and H.P.) reviewed the titles and abstracts for all citations to identify studies that met inclusion criteria. If the reviewers could not determine from the abstract whether a particular study met inclusion criteria, the article advanced to a full-text review. Articles that were selected for inclusion based on the title and abstract also advanced to full-text review.

### Data extraction

Three investigators (L.Y., S.T., and R.L.) independently used a standardized data extraction form to collect the following: first author name, publication year, country and setting (community, outpatient clinics, inpatient), participant characteristics (age, study inclusion criteria), sample size, study design, years of study, depression screening tool (PHQ-2 and/or PHQ-9), gold standard comparison, screening tool and gold standard administration protocol (e.g., timing and blinding procedures), outcome measures, and main results (with a focus on biometric properties and internal consistency). For any missing data, if valid author contact information was available, we reached out directly to request information, allowing for a response time of 2 months. Data from articles published only in Chinese were abstracted by two bilingual investigators (L.Y. and R.L.).

### Biometrics and meta-analysis

Sensitivity and specificity from the studies were combined in random effects meta-analyses separated by PHQ-9 and PHQ-2. Subgroups within these groups were analyzed by the ideal cutoff for the questionnaires (a cutoff of 10 for PHQ-9 and a cutoff of 3 for PHQ-2). Results are presented in forest plots with the random pooled effect size (sensitivity or specificity) and 95% confidence bounds. As studies did not provide complete sets of their original data, a meta-analysis could not be performed on the Cronbach's alpha or area under the curve (AUC). We, therefore, present a range of Cronbach's alpha and AUCs for the PHQ-9 and PHQ-2 for all included studies, as well as test/retest reliability for those studies with this information.

### Quality assessment

Two investigators (L.Y. and S.T.) independently assessed the methodological quality of the studies using the QUADAS-2, a tool specifically developed to assess the quality of diagnostic accuracy studies included in systematic reviews. The tool investigates potential for bias in four domains: (1) patient selection, (2) index test, (3) reference standard (including blinding), and (4) flow and timing. As recommended by the tool development team,^34^ we made several modifications according to relevance to our research question, and classified each study as overall low, high, or indeterminate risk for bias after taking all four domains into consideration. For the full version of our modified QUADAS-2, please see Appendix A2. This systematic review is considered exempt by the University of California, San Francisco IRB criteria.

## Results

### Study characteristics

**FIG. 1.**
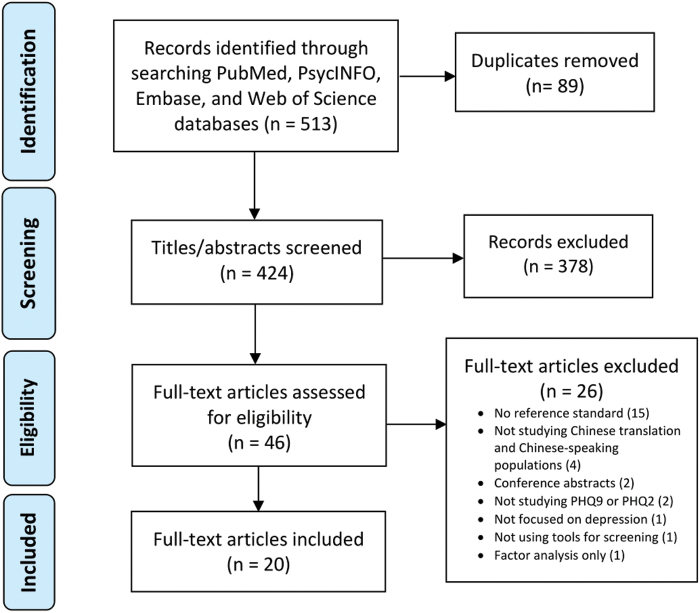
PRISMA flow diagram of study selection.

Our search strategy yielded 513 articles, of which 20 were included (see Fig. 1 for PRISMA flow diagram). Table 1 summarizes the characteristics of the included studies. As we were looking specifically for screening tool validation studies, all included studies had a cross-sectional design. Six studies^35–39^ were available only in Chinese; data were abstracted and translated by our team (L.Y., R.L.) for analysis. Seventeen studies were set in mainland China, one in Hong Kong, one in Taiwan, and one in the United States.

**Table 1.** Description and Results of Included Studies

Study	Administration	Gold standard	Setting	Participants	Biometric properties	Cronbach's α^a^	Blinding^b^/limitations	Risk of bias
PHQ-9								
Wang et al. (2014)^49^	Self	MINI; within 2 weeks	Mainland China; urban community; 2011–2012	1045; adults 16 and over	Ideal cutoff value: 7 Sensitivity: 0.86 Specificity: 0.86 AUC^b^: 0.92 [0.86–0.97] Test/retest correlation coefficient (2 weeks): 0.86^c^	0.86	Blinding is unknown.	L
Yuan (2019)^43^	Self	MINI	Mainland China; hospital; 2013–2015	782; adult patients in the coronary care unit with diagnosis of Acute Coronary Syndrome	Ideal cutoff value: 10 Sensitivity: 0.869 Specificity: 0.847 AUC: 0.842 [0.806–0.894] Test/retest correlation coefficient: not reported	0.837	Blinded. Administration of gold standard relative to index is unknown.	I
Yeung et al. (2008)^25^	Self	bCB-SCID-I/P; within 4 weeks	United States; Urban outpatient clinics; 2004–2005	1940; adults 18 and over; Chinese Americans	Preset cutoff value: 15 Sensitivity: 0.81 Specificity: 0.98 AUC: 0.97 Test/retest correlation coefficient: Not reported	0.91	Not blinded.	L
Zhang et al. (2013)^42^	Self	MINI	Hong Kong; outpatient; 2010–2011	586; Chinese patients between 25 and 75 years of age with type 2 diabetes	Ideal cutoff value: 7 Sensitivity: 0.826 Specificity: 0.737 AUC: 0.85 [0.76–0.94] Test/retest correlation coefficient (2–4 weeks): 0.70^d^	0.86	Blinding is unknown. Administration of gold standard relative to index is unknown.	L
Ye et al. (2020)^44^	Self, with personnel guidance if needed	American DSM-V; immediately	Mainland China; outpatient and inpatient; 2018	148; patients with psoriasis	Ideal cutoff value: 9 Sensitivity: 0.98 Specificity: 0.908 AUC: 0.979 [0.968–0.991] Test/retest correlation coefficient (1 week): 0.955^c^	0.938	Blinding is unknown. Exclusion criteria are unclear.	I
Chen et al. (2013)^46^	Self	SCID; within 2 weeks	Mainland China; primary care clinics; 2009–2010	2639; adult patients 18 and over	Ideal cutoff value: 10 Sensitivity: 0.87 Specificity: 0.81 AUC: 0.91 [0.87–0.94] Test/retest correlation coefficient (2 weeks): 0.76^c^	0.89	Blinded. 7% dropout rate.	L
Du et al. (2017)^48^	Self (online)	MINI; within 48 h	Mainland China; university	230; adult university students 18 and over	Ideal cutoff value: 10 Sensitivity: 0.74 Specificity: 0.85 AUC: 0.897 [0.823–0.970] Test/retest correlation coefficient (2 weeks): 0.78^e^	0.80	Blinded.	L
Peng et al. (2020)^45^	Self	SCID	Mainland China; outpatient; 2019	258; adults with acne between ages 18 and 24	Preset cutoff value: 9 Sensitivity: 0.957 Specificity: 0.886 AUC: 0.973 [0.956–0.990] Test/retest correlation coefficient (1 week): 0.824^c^	0.851	Blinding is unknown. Administration of gold standard relative to index is unknown. 8% dropout rate.	I
PHQ-2 and PHQ-9								
Liu et al. (2016)^40^	By postgraduate students and psychiatrists	SCID-I; immediately	Mainland China; rural community; 2010–2011	839; adults 60 and over	Ideal cutoff value for PHQ-9: 8 Sensitivity: 0.97 Specificity: 0.89 AUC: 0.97 [0.96–0.98] Test/retest correlation coefficient for PHQ-9: Not reported Ideal cutoff value for PHQ-2: 3 Sensitivity: 0.90 Specificity: 0.90 AUC: 0.94 [0.90–0.97] Test/retest correlation coefficient for PHQ-2: Not reported	PHQ-9: 0.82 PHQ-2: 0.76	Not blinded.	L
Xiong (2015)^47^	Self	MINI	Mainland China; outpatient clinics; 2011–2012	491; adults 18 and over with multiple somatic symptoms	Ideal cutoff value for PHQ-9: 10 Sensitivity: 0.77 Specificity: 0.76 AUC: 0.82 [0.77–0.86] Test/retest correlation coefficient for PHQ-9: not reported Ideal cutoff value for PHQ-2: 3 Sensitivity: 0.77 Specificity: 0.74 AUC: 0.81 [0.77–0.86] Test/retest correlation coefficient for PHQ-2: not reported	0.90	Blinding is unknown. Administration of gold standard relative to index is unknown.	I
Zhang et al. (2013)^50^	Self	SCID; within 2 weeks	Mainland China; University	959; Chinese college students	Ideal cutoff value for PHQ-9: 11 Sensitivity: 0.89 Specificity: 0.97 AUC: 0.977 [0.966–0.988] Test/retest correlation coefficient (4 weeks) for PHQ-9: 0.873^f^ Ideal cutoff value for PHQ-2: 3 Sensitivity: 0.81 Specificity: 0.96 AUC: 0.939 [0.911–0.967] Test/retest correlation coefficient (4 weeks) for PHQ-2: 0.829c	PHQ-9: 0.854 PHQ-2: 0.727	Blinded. 13.8% dropout rate.	L
Xia (2019)^51^	Self	MINI; within 1 day	Mainland China; outpatient, 2018	213; Chinese adult patients with epilepsy	Ideal cutoff value for PHQ-9: 7 Sensitivity: 0.8286 Specificity: 0.8427 AUC: 0.888 [0.838–0.927] Test/retest correlation coefficient for PHQ-9: Not reported Ideal cutoff value for PHQ-2: 2 Sensitivity: 0.7714 Specificity: 0.7528 AUC: 0.802 [0.742–0.853] Test/retest correlation coefficient for PHQ-2: not reported	PHQ-9: 0.86	Blinded.	L
Chen (2010)^53^	Self, with assistance from nurse as needed	SCID	Mainland China; primary care clinics; 2008	364; adult patients 60 and over	Ideal cutoff value for PHQ-9: 9 Sensitivity: 0.86 Specificity: 0.85 AUC: 0.92 [0.88–0.96] Test/retest correlation coefficient for PHQ-9: not reported Ideal cutoff value for PHQ-2: 3 Sensitivity: 0.84 Specificity: 0.90 AUC: 0.92 [0.87–0.97] Test/retest correlation coefficient for PHQ-2: not reported	PHQ-9: 0.91	Blinded. Administration of gold standard relative to index is unknown. 20% dropout rate.	L
Liu et al. (2011)^41^	Self	SCAN	Taiwan; community-based primary care clinics; 2007–2008	1954; adult patients 18 and over	Ideal cutoff value for PHQ-9: 10 Sensitivity: 0.86 Specificity: 0.939 AUC: 0.96 [0.93–0.98] Test/retest correlation coefficient (2 weeks): 0.87^g^ Ideal cutoff value for PHQ-2: 2 Sensitivity: 0.88 Specificity: 0.82 AUC: 0.90 [0.85–0.95] Test/retest correlation coefficient for PHQ-2: not reported	PHQ-9: 0.80	Blinded. Administration of gold standard relative to index is unknown. 21.6% dropout rate.	L

Studies published in Chinese language journals								
PHQ-9								
Jin et al. (2011)^39^	Self	SCID	Mainland China; community	1275; adult patients 60 and over	Preset cutoff value: 10 Sensitivity: 0.8831 Specificity: 0.825 AUC: not reported Test/retest correlation coefficient: not reported	0.767	Blinding is unknown. Administration of gold standard relative to index is unknown.	I
Xu et al. (2007)^38^	Self, with guidance from family or research staff if illiterate	SCID; within 1 week	Mainland China; community	622; adult patients 60 and over	Preset cutoff value: 15 Sensitivity: 0.88 Specificity: 0.99 AUC: not reported Test/retest correlation coefficient for PHQ-9 (1 week): 0.934	0.8325	Blinding is unknown.	I
Yang et al. (2015)^37^	Self	SCID	Mainland China; outpatient	258; psychosomatic outpatients between 16 and 65	Preset cutoff value: 10 Sensitivity: 0.98 Specificity: 0.67 AUC: not reported Test/retest correlation coefficient: not reported	Not reported	Blinded. Administration of gold standard relative to index is unknown.	L
Zhi et al. (2013)^36^	Self	MINI; immediately	Mainland China; outpatient	2009; adult patients between 18 and 65	Ideal cutoff value: 8 Sensitivity: 0.856 Specificity: 0.802 AUC: 0.903 [0.869–0.937] Test/retest correlation coefficient: not reported	0.855	Blinding is unknown. 0.6% dropout rate.	L
Chen et al. (2015)^35^	Self, with personnel guidance if needed	MINI; immediately	Mainland China; outpatients Department of Psychiatry; 2012–2013	319; adult patients 18 and over	Cutoff values reported: 6 (mild); 12 (moderate); 15 (severe) Sensitivity: not reported for mild depression; 0.94; 0.83 Specificity: not reported for mild depression; 0.82; 0.90 AUC: 0.94 Test/retest correlation coefficient: not reported	0.89	Blinding is unknown.	L
PHQ-2 and PHQ-9								
Wang et al. (2015)^33^	Self	CIDI; immediately	Mainland China; outpatients from Psycho-cardiological Department; 2013–2014	201; Adult patients 18 and over	Ideal cutoff value for PHQ-9: 10 Sensitivity: 0.871 Specificity: 0.835 AUC: 0.877 Test/retest correlation coefficient for PHQ-9 (1 week): 0.882^h^ Ideal cutoff value for PHQ-2: 2 Sensitivity: 0.857 Specificity: 0.692 AUC: 0.806 Test/retest correlation coefficient for PHQ-2 (1 week): 0.813e	PHQ-9: 0.809 PHQ-2: 0.785	Blinded	L

^a^ As a measurement of internal consistency.

^b^ “Blinded” refers to the interviewers not knowing the PHQ-2 and PHQ-9 scores before conducting the clinical interview.

^c^ Conducted after 2 weeks.

^d^ Conducted after 2 weeks on 6.82% of the participants (40/586).

^e^ Conducted after 2 weeks on 65.2% of the participants (150/230).

^f^ Conducted after 4 weeks on 10% of the participants (121/959).

^g^ Conducted after 2 weeks on 13.1% of the participants (256/1954).

^h^ Conducted after 1 week on 24.9% of the participants (50/201).

ACS, Acute Coronary Syndrome; AUC, area under the curve; bCB, Chinese Bilingual version of the PHQ-9; CIDI, Composite International Diagnostic Interview; DSM-V, *American Diagnostic and Statistical Manual of Mental Disorders*, fifth edition; I, indeterminate; L, low; MINI, Mini International Neuropsychiatric Interview; PHQ, Patient Health Questionnaire; SCAN, Schedule for Clinical Assessments in Neuropsychiatry; SCID, Structured Clinical Interview for Diagnostic and Statistical Manual Disorders.

Across studies, we observed a wide range in sample size (*n*=148–2639) as well as clinical setting (primary care vs. specialty outpatient care vs. hospital inpatients). Samples ranged from patients with specific medical conditions such as cardiac disease or psoriasis, to general primary care patients, to individuals in the community. All samples consisted of patients who were Chinese speaking only, except for the study set in the United States. That study stated that the majority of their patient population were “less acculturated Chinese immigrants,” although they did not identify the proportion of their sample that truly had LEP.^25^

All 20 included studies examined the PHQ-9. Of these, Yeung et al utilized a bilingual (English and Chinese) PHQ-9, which the investigator team translated themselves.^25^ All other teams examined only the Chinese language PHQ-9; Liu et al^40^ utilized the Chinese portion of Yeung et al questionnaire, Liu et al^41^ used their own translation, Zhang et al reported that they used a translation available on the Hong Kong government website,^42^ and the remaining studies did not specify the version of the Chinese PHQ-9 used.

For their gold standard, nine of our included studies used the SCID, eight used the MINI, one used the SCAN, one used the CIDI, and one diagnosed subjects according to the *American Diagnostic and Statistical Manual of Mental Disorders*, Fifth Edition criteria (unclear if they relied on SCID).^44^ For all studies, except one, the PHQ-9 was completed before the gold standard (therefore blinded to the results of the clinical interview); the remaining article, Chen et al,^35^ did not specify the details of the study protocol and we could not confirm the details by reaching out to the investigators. For 13 studies, we were able to identify the intervals between conducting the PHQ-9 and the gold standard, which ranged from immediate to four weeks. We were able to confirm that investigators conducting the gold standard were blinded to PHQ-9 results for 10 studies. For eight studies, only a select subset of the study sample (selected *a priori*) was asked to complete the gold standard for comparison.

### Biometrics and meta-analysis

For the Chinese language PHQ-9, internal consistency varied across studies, with the Cronbach's alpha ranging from 0.765 to 0.938. Five studies preset a cutoff value and calculated the Chinese language PHQ-9 sensitivity/specificity using that cutoff value—two studies chose 10 as the cutoff,^37,39^ two studies chose 15,^25,38^ and one study chose 9.^45^ Studies that did not use a preset cutoff value and conducted receiver operating characteristic (ROC) curve analyses found that the area under the ROC curve ranged from 0.78 to 0.977. These studies identified ideal cutoff values ranging from 6 to 11, with 10 being the most common (6/15 studies).

**FIG. 2.**
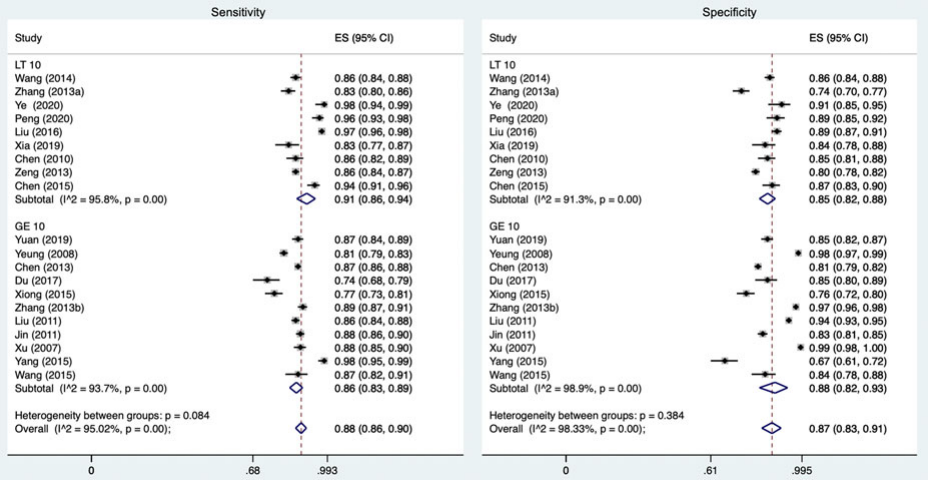
Meta-analyses of sensitivity (*left*) and specificity (*right*) of PHQ-9 by cutoff score. ES, effect size; LT, less than; GE, greater than or equal to; I^2=variation in effect size attributable to heterogeneity. PHQ, Patient Health Questionnaire.

For the nine studies that identified or preset a cutoff value of less than 10, the meta-analysis demonstrated a pooled sensitivity of 0.91 (95% CI 0.86–0.94) and a pooled specificity of 0.85 (95% CI 0.82–0.88; see Fig. 2). For the eleven studies that identified or preset a cutoff value of greater than or equal to 10, the pooled sensitivity was 0.86 (95% CI 0.83–0.89) and the pooled specificity was 0.88 (95% CI 0.82–0.93). Overall, the pooled sensitivity of studies evaluating the Chinese language PHQ-9 was 0.88 (95% CI 0.86–0.90), and the pooled specificity was 0.87 (95% CI 0.83–0.91).

Ten studies additionally analyzed the test/retest reliability for the Chinese language PHQ-9. Of these, four retested their patients after an interval of 1 week, resulting in coefficients ranging from 0.824 to 0.955^38,44,45^; five retested their patients after 2 weeks, resulting in coefficients ranging from 0.70 to 0.87^41,42,46,48,49^; and one study retested their patients after 4 weeks, resulting in a coefficient of 0.873.^50^

### Patient health questionnaire-2

**FIG. 3.**
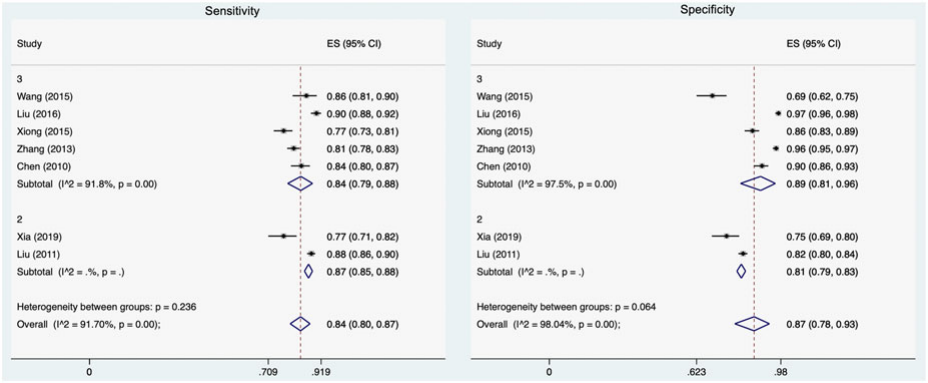
Meta-analyses of sensitivity (*left*) and specificity (*right*) of PHQ-2 by cutoff score.

Seven of our included studies used a subset of their data to examine the Chinese language PHQ-2. Cronbach's alpha ranged from 0.727 to 0.785. The area under the ROC curve (AUC) ranged from 0.802 to 0.94. Five studies identified 3 as the ideal cutoff score; at this cutoff, the pooled sensitivity was 0.84 (95% CI 0.79–0.88) and the pooled specificity was 0.89 (95% CI 0.81–0.96; Fig. 3). For the two remaining studies that identified the ideal cutoff value as 2, the pooled sensitivity was 0.87 (95% CI 0.85–0.88) and the pooled specificity was 0.81 (95% CI 0.79–0.83). Overall, the pooled sensitivity of studies evaluating the Chinese language PHQ-2 was 0.84 (95% CI 0.80–0.87), and the pooled specificity was 0.87 (95% CI 0.78–0.93).

Only two studies evaluated the test/retest reliability for the Chinese language PHQ-2. One study evaluated the reliability after 1week, resulting in a coefficient of 0.813; another study evaluated the reliability after 4 weeks, resulting in a coefficient of 0.829.^42^

### Quality assessment

After assessment with our modified QUADAS-2 tool, none of the included studies had a high risk of bias (Table 2). Twelve included studies had a low risk of bias, while eight studies had an indeterminate risk of bias, attributed to missing key information, including whether study team members conducted the PHQ while blinded to the gold standard results or vice versa. In particular, Ye et al^44^ did not describe their exclusion criteria when enrolling patients; this limited our ability to evaluate, for example, how much of their sample had pre-existing psychiatric illness that would invoke bias when studying the efficacy of a depression screening tool.

**Table 2.** Quality Assessment Details of Included Studies

Question	Wang et al. (2014)^49^	Yuan (2019)^43^	Yeung et al. (2008)^25^	Zhang et al. (2013)^50^	Ye et al. (2020)^44^	Chen et al. (2013)^46^	Du et al. (2017)^48^
Overall risk of bias	Low	Indeterminate: unknown interval between test and reference standard	Low	Low	Indeterminate: no description of exclusion criteria, unknown blinding protocol	Low	Low
Was a consecutive or random sample of patients enrolled?	Yes: probability proportionate to size sampling and simple random sampling	Yes: consecutive	Yes: consecutive	Yes: consecutive	Yes: consecutive	Yes: random	University student email list—unclear if truly random
Was a case/control design avoided?	Yes	Yes	Yes	Yes	Yes	Yes	Yes
Did the study avoid inappropriate exclusions?	Yes	Yes: illness preventing completion, previous history of mental illness or psychotherapy, unwilling	Yes	Yes: disabling disease or reduced life expectancy, difficulty communicating	Information not found	Yes: patients with psychotic disorders	Yes
Were the index test^a^ results interpreted without knowledge of the results of the reference standard^b^?	Yes	Yes	Yes	Yes	Yes	Yes	Yes
Are the specificity and sensitivity recorded for multiple cutoff scores?	Yes	Yes	No: only one (15)	Yes	Yes	Yes	Yes
Was the index test administered in a standardized fashion?	Yes	Yes	Yes	Yes	Yes	Yes	Yes
Was an appropriate version of the index test used?	Information not found	Information not found	Yes: translated by investigators	Yes: translation on Hong Kong government website	Information not found	Information not found	Information not found
Is the reference standard likely to correctly classify the target condition?	Yes: MINI	Yes: MINI	Yes: SCID	Yes: MINI	Yes: DSM-V	Yes: SCID	Yes: MINI
Were the reference standard results interpreted without knowledge of the results of the index test?	Information not found	Yes	No: SCID conducted for all patients with PHQ-9≥15 and 8% of patients with PHQ-9<15	Yes	Information not found	Yes	Yes
Was the reference standard administered in a standardized fashion?	Yes: one interviewer	Yes: standardized training	Yes: one interviewer	Yes	Yes: one interviewer	Yes: two psychiatrists with interrater reliability 0.81	Yes: two psychiatrists
Was there an appropriate interval between index tests and reference standard?	Yes: <2 weeks	Information not found	No: “about 4 weeks”	No: “within 2–4 weeks”	Yes: immediate	Yes: 2 weeks	Yes: within 48 h
Did all patients receive a reference standard?	Yes	Yes	No: subsample analysis	No: subsample analysis	Yes	No: subsample analysis	No: subsample analysis
Did patients receive the same reference standard?	Yes	Yes	Yes	Yes	Yes	Yes	Yes
Were all patients included in the analysis without a significant percentage of dropouts?	Yes: 0% dropout rate (1045/1045)	Yes: 0% dropout rate (782/782)	Yes: 0% dropout rate (1940/1940)	Yes: 0% dropout rate (586/586)	Yes: 0% dropout rate (148/148)	Yes: 7% dropout rate (280/300)	Yes: 0% dropout rate (230/230)

Question	Peng et al. (2020)^45^	Liu et al. (2016)^40^	Xiong (2015)^47^	Zhang et al. (2013)^50^	Xia (2019)^51^	Chen (2010)^53^	Liu et al. (2011)^41^
Overall risk of bias	Indeterminate: unknown blinding protocol, interval between test and reference standard	Low	Indeterminate: unknown blinding protocol, interval between test and reference standard	Low	Low	Low	Low
Was a consecutive or random sample of patients enrolled?	Yes: consecutive	Yes: study province → random towns → random villages → all individuals	Yes: on randomly assigned days, patients consecutively invited	Yes	Yes: consecutive	Yes: consecutive	Yes: consecutive
Was a case/control design avoided?	Yes	Yes	Yes	Yes	Yes	Yes	Yes
Did the study avoid inappropriate exclusions?	Yes: patients with pre-existing mental illness, severe physical illness	Yes: if not in area during study period or difficulty with communication due to illness	Yes: wrong language, limited writing skills, cognitive impairment, psychosis, acute suicidality	Yes	Yes: severe psychiatric disorders or antisocial, schizotypal personality disorders	Yes	Yes
Were the index test^a^ results interpreted without knowledge of the results of the reference standard^b^?	Yes	Yes	Yes	Yes	Yes	Yes	Yes
Are the specificity and sensitivity recorded for multiple cutoff scores?	No: only one (9)	Yes	Yes	Yes	Yes	Yes	Yes
Was the index test administered in a standardized fashion?	Yes	Yes	Yes	Yes	Yes	Yes	Yes
Was an appropriate version of the index test used?	Information not found	Yes: cited Yeung et al. 2008	Information not found	Information not found	Information not found	Information not found	Yes: study team translated and back translated
Is the reference standard likely to correctly classify the target condition?	Yes: SCID	Yes: SCID	Yes: MINI	Yes: SCID	Yes: MINI	Yes: SCID	Yes: SCAN
Were the reference standard results interpreted without knowledge of the results of the index test?	Information not found	No: SCID conducted immediately after PHQ-9 by same interviewer	Information not found	Yes	Yes	Yes	Yes
Was the reference standard administered in a standardized fashion?	Yes: two psychiatrists	Yes: standardized interview training	Yes	Yes: standardized training	Yes: one interviewer	Yes: two psychiatrists	Yes: trained staff with 0.88 interrater reliability
Was there an appropriate interval between index tests and reference standard?	Information not found	Yes: immediate	Information not found	Yes: <2 weeks	Yes: <24h	Information not found	Information not found
Did all patients receive a reference standard?	Yes	Yes	Yes	Yes	Yes	No: subsample analysis	Yes
Did patients receive the same reference standard?	Yes	Yes	Yes	Yes	Yes	Yes	Yes
Were all patients included in the analysis without a significant percentage of dropouts?	Yes: 8% dropout rate (275/300)	Yes: 0.7% dropout rate (839/845)	Yes: 0% dropout rate (491/491)	Significant: 13.8% dropout rate (959/1112)	Yes: 0% dropout rate (213/213)	Significant: 20% dropout rate (77/96) for subsample chosen to undergo reference standard	Significant: 21.6% dropout rate, however, sample was large (1532/1954)

Question	Jin et al. (2011)^39,c^		Xu et al. (2007)^38,c^	Yang et al. (2015)^37,c^	Zhi et al. (2013)^36,c^	Chen (2015)^35,c^	Wang et al. (2015)^33,c^
Overall risk of bias	Indeterminate: unknown blinding protocol, interval between test and reference standard		Low	Low	Low	Low	Low
Was a consecutive or random sample of patients enrolled?	Yes: random		Yes: random	Yes: consecutive	Yes: consecutive	Yes	Yes: consecutive
Was a case/control design avoided?	Yes		Yes	Yes	Yes	Yes	Yes
Did the study avoid inappropriate exclusions?	Yes: dementia or physically incapable of completing questionnaire		Yes	Yes: incapable of understanding and responding to questionnaire, severe mental health symptoms or physical disability, substance use disorders, currently receiving medication or therapy treatment for mental illness	Yes: physically incapable of completing questionnaire, severe physical or mental illness	Yes: those incapable of completing study or consent due to language or hearing	Yes
Were the index test results interpreted without knowledge of the results of the reference standard?	Yes		Yes	Yes	Yes	Information not found	Yes
Are the specificity and sensitivity recorded for multiple cutoff scores?	No: only one (10)		No: only one (15)	No: only one (10)	Yes	Yes	Yes
Was the index test administered in a standardized fashion?	Yes		Yes	Yes	Yes	Yes	Yes
Was an appropriate version of the index test used?	Information not found		Information not found	Information not found	Information not found	Information not found	Yes: cited Liu 2011's translation
Is the reference standard likely to correctly classify the target condition?	Yes: SCID		Yes: SCID	Yes: SCID	Yes: MINI	Yes: MINI	Yes: CIDI
Were the reference standard results interpreted without knowledge of the results of the index test?	Information not found		Information not found	Yes	Information not found	Information not found	Yes
Was the reference standard administered in a standardized fashion?	Yes: standardized training		Yes: two psychiatrists	Yes: standardized training	Yes: standardized training	Yes	Yes: one interviewer
Was there an appropriate interval between index tests and reference standard?	Information not found		Yes: 1 week	Information not found	Yes: immediate	Yes: immediate	Yes: immediate
Did all patients receive a reference standard?	No: subsample analysis		No: subsample analysis	No: subsample analysis	Yes	Yes	Yes
Did patients receive the same reference standards?	Yes		Yes	Yes	Yes	Yes	Yes
Were all patients included in the analysis without a significant percentage of dropouts?	Yes: 0% dropout rate (117/117)		Yes: 0% dropout rate (195/195)	Yes: 0% dropout rate (97/97)	0.6% dropout rate (1997/2009)	Yes: 0% dropout rate (319/319)	Yes: 0% dropout rate (201/201)

^a^ Index test=PHQ.

^b^ Reference standard=old standard clinical interview used by the particular study.

^c^ Study published in Chinese.

## Discussion

In this systematic review, we found that the available literature supports the use/validity of the Chinese PHQ-9 and PHQ-2 as a tool for screening for depression in monolingual Chinese patients. We found high sensitivity and specificity for depression for both the PHQ-2 and PHQ-9 among individuals who spoke Chinese languages, across a variety of clinical settings and with a range of clinical comorbidities. Our findings are consistent with the two previous systematic reviews that have been conducted in this area. Our review has unique strengths, including a greater number of studies, comparison to gold standard clinical interviews as an inclusion criterion, studies encompassing broad geographic settings and patient populations and, therefore, better generalizability, and examination of both English- and Chinese-language articles.

The studies included in our review that evaluated the validity of the Chinese PHQ-9 at multiple cutoff scores identified different ideal cutoffs, ranging from 6 to 11, with 10 identified as the optimal cutoff score in 6 of 15 studies. This is consistent with how the PHQ-9 is currently used in primary care settings, with a score of 10 as the cutoff for a positive screen across languages.^21^ Notably, it is also comparable to the English language PHQ-9 at this cutoff.^52^ However, not all studies in our review agreed on this cutoff, with many identifying lower scores as the optimal cutoff for diagnosing depression. This points to the need for further investigation to ensure that we are not missing depression in Chinese patients with LEP, who are already at high risk of depression under-recognition and undertreatment.

Additionally, the English language PHQ-9 can also be used to evaluate symptom severity, with scores of 5, 10, 15, and 20 indicating mild, moderate, moderately severe, and severe depression, respectively.^21^ Of the studies we found, only Chen et al identified score cutoffs for different levels of symptom severity: 6, 12, and 15 for mild, moderate, and severe depression.^34^ Yeung et al indirectly acknowledged this by setting the cutoff for a positive screen at the higher score of 15 instead of 10, to identify subjects whose depression was significant enough to warrant treatment; Xu et al also set their cutoff at 15 and did not state their justification, but presumably had similar reasoning.^25,38^ Although our review did not explicitly address this question, for providers who wish to use the PHQ-9 to monitor response to treatment, further research could help confirm ideal cutoffs for depression symptom severity.

Less than half the articles we found evaluated the PHQ-2 in addition to the PHQ-9; all seven of these articles validated the PHQ-2 as a screening tool, with four out of seven studies agreeing on 3 as the best cutoff value for screening positive for depression, as is used for the English language PHQ-2.

We recognize several limitations to our systematic review. First, despite a rigorous search for relevant articles, it is possible that some were missed; in particular, although we were able to include six Chinese language studies that were identified through our search, we did not specifically examine the Chinese language literature or databases and may have missed studies that were published only in Chinese. However, as our purpose is to apply these findings to monolingual Chinese speakers in the United States, we felt it was appropriate to limit to Chinese language articles in English language databases for this review.

Second, we did not target any specific practice setting for our search; our ability to make strong recommendations for clinicians may thus be limited by the variability in patient comorbidities or countries of residence among the included studies. However, the broad range of populations represented in our review improves generalizability for the PHQ-2/9 as a broad screening tool. Third, although our search was internationally targeted, most studies that fit our inclusion criteria were conducted in mainland China.

Although a single study was conducted in the United States, Yeung et al, similarly found high sensitivity and specificity, the dearth of studies around the use of the Chinese PHQ in settings with patient/provider language discordance points to the need for more research in this direction. In the United States, while Asian Americans account for 5.7% of the population, less than 1% of National Institutes of Health funding goes to research on Asian American health.^54^ Additionally, for immigrant populations, the preferred language is frequently used as a measure of acculturation^55^; U.S. patients preferring the Chinese language PHQ are therefore more likely to be recent immigrants and/or less acculturated to the United States, implying some crossapplicability to research conducted with nondiasporic Chinese patients. Fourth, the quality of the diagnoses made through clinical interviews may vary depending on the individual investigator and the specific clinical interview used, which could affect the internal validity of our included studies.

Fifth, as the PHQ-2 and PHQ-9 are usually given as written questionnaires, we did not choose to distinguish between the various dialects of spoken Chinese (as all literate speakers read the same written form). However, in studies where some questionnaires were verbally administered by a research assistant, variation between the spoken dialects may have impacted tool validity. Finally, although we contacted study authors when possible to inquire about missing information, in several cases, we were unable to ascertain the exact translation of the Chinese PHQ-9 or PHQ-2 used, whether the studies appropriately excluded patients with pre-existing psychiatric illness, or whether the investigators were double blinded to the PHQ and gold standard results. This particularly impacted our evaluation of the six studies published only in Chinese, which did not provide contact information for the study authors.

## Conclusion

Chinese patients with LEP and depression are more likely to be underdiagnosed and undertreated, leading to worse health outcomes and quality of life. As the mental health burden for the Asian American community has increased during the COVID-19 pandemic with the rise in racism and violence, it is more urgent than ever for us to ensure we are using the right tools to identify patients with depression. Despite the limitations of our review, we found strong evidence supporting the accuracy of Chinese language versions of the PHQ-9 and PHQ-2 for screening for depression across practice settings. However, studies reported a wide range of cutoff scores for the PHQ-9, with many demonstrating high sensitivity and specificity at lower cutoff scores, alluding to the possibility that the ideal cutoff score for Chinese monolingual patients may differ from the score used for English speakers. If so, the PHQ-9 as currently used in practice may miss depressive symptoms in some Chinese monolingual patients.

To effectively address mental health disparities for patients with LEP in the United States, more research is necessary to investigate this possibility specifically among Chinese monolingual patients living in the United States and to establish the validity of depression screening tools in other commonly spoken non-English languages. Finally, once the research is robust, medical institutions and professional bodies must standardize the uptake of evidence-based depression screening tools and interventions to truly impact patient care.

## Abbreviations Used

SCID: Structured Clinical Interview for DSM
MINI: Mini-International Neuropsychiatric Interview
CIDI: Composite International Diagnostic Interview
SCAN: Schedules for Clinical Assessment in Neuropsychiatry
AUC: area under the curve
DSM-V: American Diagnostic and Statistical Manual of Mental Disorders, Fifth Edition
ROC: receiver operating characteristic
PHQ: Patient Health Questionnaire
LEP: limited English proficiency

## Appendix

**Appendix A1.** PHQ-9 PHQ-2 Questionnaire Chinese: Search Appendix

DATABASE	SEARCH STRATEGY
PubMed	(“PHQ-9” OR “PHQ-2” OR “patient health questionnaire-9” OR “patient health questionnaire-2” OR “Patient Health Questionnaire”[Mesh]) AND (“Depression/diagnosis”[Mesh] OR depression screening OR depression assessment OR “Depressive Disorder/diagnosis”[Mesh] OR depressive disorder screening OR depressive disorder assessment) AND (efficacy OR reliability OR validity OR utility OR “Validation Studies as Topic”[Mesh]) AND (Cantonese OR Mandarin OR Vietnamese OR Chinese OR China OR “China”[Mesh] OR Taiwan OR “Taiwan”[Mesh] OR Vietnam OR “Vietnam”[Mesh])
Web of Science	(“PHQ-9” OR “PHQ-2” OR “patient health questionnaire-9” OR “patient health questionnaire-2”) AND (“Depression diagnosis” OR depression screening OR depression assessment OR depressive disorder screening OR depressive disorder assessment) AND (efficacy OR reliability OR validity OR utility) AND (Cantonese OR Mandarin OR Vietnamese OR Chinese OR China OR Taiwan OR Vietnam)
Embase	(‘patient health questionnaire 2′/exp OR ‘patient health questionnaire 2′ OR ‘patient health questionnaire 9′/exp OR ‘patient health questionnaire 9′) AND (‘depression’/exp/dm_di OR ‘depression assessment’ OR ‘depression screening’) AND (‘efficacy parameters’/exp OR ‘efficacy parameters' OR ‘efficacy’/exp OR efficacy OR ‘validity’/exp OR validity OR ‘reliability’/exp OR reliability OR utility) AND (‘chinese’/exp OR chinese OR ‘cantonese language’/exp OR ‘cantonese language’ OR ‘cantonese’/exp OR cantonese OR ‘mandarin’/exp OR mandarin OR ‘mandarin language’/exp OR ‘mandarin language’ OR ‘vietnamese’/exp OR Vietnamese OR ‘china’/exp OR china OR ‘viet nam’/exp OR ‘viet nam’ OR ‘taiwan’/exp OR taiwan)
PsycINFO	(“PHQ-9” OR “PHQ-2” OR “patient health questionnaire-9” OR “patient health questionnaire-2”) AND (“Depression diagnosis” OR depression screening OR depression assessment OR depressive disorder screening OR depressive disorder assessment) AND (efficacy OR reliability OR validity OR utility) AND (Cantonese OR Mandarin OR Vietnamese OR Chinese OR China OR Taiwan OR Vietnam)

### Appendix A2. QUADAS 2 Tool

*Modified by Leena Yin & Dr. Maria Garcia*

This tool is a modified version of the QUADAS 2 (https://www.bristol.ac.uk/population-health-sciences/projects/quadas/quadas-2/), developed by a team led by the University of Bristol as a method of assessing quality and risk of bias in systematic reviews. Major modifications for our review included the following:

-The original QUADAS 2 included two parts under each domain: part 1 addressing internal validity, and part B addressing external validity. As we have selected our studies carefully based on applicability to our study population as part of the search criteria, we felt it reasonable to remove part 2 from this portion of the analysis. Considerations for external validity will be included in the discussion.
-Three questions were added, and one was modified, based on applicability to our review.
-Taking each of the four domains into consideration, we classified each study as overall low, high, or unclear risk for bias.

*For all main questions, respond with low/high/unclear risk*

*For all subquestions, respond with yes/no/unclear*

**Domain 1: Patient Selection**

**Could the selection of patients have introduced bias?**

A. Was a consecutive or random sample of patients enrolled?
B. Was a case-control design avoided?
C. Did the study avoid inappropriate exclusions?

**Domain 2: Index Tests**

**Could the conduct or interpretation of the index test have introduced bias?**

A. Were the index test results interpreted without knowledge of the results of the reference standard? *(may need to ask the authors)*
B. *(modified)* Are the specificity and sensitivity recorded for multiple cutoff scores?
C. *(new)* Was the index test administered in a standardized fashion?
D. *(new)* Was an appropriate version of the index test used?

**Domain 3: Reference Standard**

**Could the reference standard, its conduct, or its interpretation have introduced bias?**

A. Is the reference standard likely to correctly classify the target condition?
B. Were the reference standard results interpreted without knowledge of the results of the index test?
C. *(new)* Was the reference standard administered in a standardized fashion?

**Domain 4: Flow and Timing**

**Could the patient flow have introduced bias?**

A. Was there an appropriate interval between index tests and reference standard *(<1 month)*?
B. Did all patients receive a reference standard?
C. Did patients receive the same reference standard?
D. Were all patients included in the analysis?
